# Exposure to Oil and Hypoxia Results in Alterations of Immune Transcriptional Patterns in Developing Sheepshead Minnows (*Cyprinodon variegatus*)

**DOI:** 10.1038/s41598-020-58171-8

**Published:** 2020-02-03

**Authors:** Maria L. Rodgers, Danielle Simning, Maria S. Sepúlveda, Sylvain De Guise, Thijs Bosker, Robert J. Griffitt

**Affiliations:** 1 Division of Coastal Sciences, School of Ocean Science and Engineering, University of Southern Mississippi, Ocean Springs, Mississippi 39564 USA; 2 Purdue University, Department of Forestry and Natural Resources, West Lafayette, Indiana, 47907 USA; 3 Department of Pathobiology and Veterinary Science, University of Connecticut, Storrs, Connecticut 06269 USA; 4 Leiden University College/Institute of Environmental Sciences (CML), Leiden University, Leiden, 2595DG The Netherlands

**Keywords:** Immunology, Environmental sciences, Marine biology

## Abstract

The area and timing of the *Deepwater Horizon* oil spill highlight the need to study oil and hypoxia exposure in early life stage fishes. Though critical to health, little research has targeted the effect of oil and hypoxia exposure on developing immune systems. To this end, we exposed sheepshead minnows (*Cyprinodon variegatus*) at three early life stages: embryonic; post-hatch; and post-larval, to a high energy water accommodated fraction (HEWAF) of oil, hypoxia, or both for 48 hours. We performed RNAseq to understand how exposures alter expression of immune transcripts and pathways. Under control conditions, the embryonic to post-hatch comparison (first transition) had a greater number of significantly regulated immune pathways than the second transition (post-hatch to post-larval). The addition of oil had little effect in the first transition, however, hypoxia elicited changes in cellular and humoral immune responses. In the second transition, oil exposure significantly altered many immune pathways (43), and while hypoxia altered few pathways, it did induce a unique signature of generally suppressing immune pathways. These data suggest that timing of exposure to oil and/or hypoxia matters, and underscores the need to further investigate the impacts of multiple stressors on immune system development in early life stage fishes.

## Introduction

The functionality of the immune system is key to organismal health and survival. Alteration of the immune system can have serious consequences, including immunosuppression or autoimmunity. The immune system is so critical to life that it starts developing very early on: for example, the innate immune system in zebrafish (*Danio rerio*) is detectable at day 1 of embryogenesis (via the appearance of macrophages)^1,2^ and embryonic zebrafish exposed to *Salmonella typhimurium* respond with induction of two matrix metalloproteinase genes (*mmp9* and *mmp13*)^3^. In addition, the zebrafish immune system is both morphologically and functionally mature 4–6 weeks after fertilization^4^. However, since fish are a highly diverse taxa, this period of development can widely vary depending upon species and environmental conditions.

The area of oiling from the 2010 *Deepwater Horizon* oil spill impacted sites where embryos and other developing fishes reside^5^. Given the fact that the immune system develops during early life stages, it is important to examine the immunological consequences of oil exposure on developing fish. In addition to contending with oil exposure, the *Deepwater Horizon* oil spill took place in areas that are prone to experiencing hypoxic conditions^6,7^. Literature has clearly established that exposure to polycyclic aromatic hydrocarbons (PAHs) (for example, via oil spills) results in reduced immune function in multiple fish species^8^, but there is also evidence of immunological effects of hypoxia exposure in fishes. For example, fish experiencing hypoxic conditions can have decreased complement activity^9^, reduced production of reactive oxygen species^10^, depressed respiratory burst activity in head kidney leukocytes^11^, and lower antibody responses^12^. Exposure to hypoxia also reduces expression of immune-related genes (*infα* and *mx*) in head kidney macrophages after stimulation with poly I:C (an immunostimulant), potentially indicating that hypoxia reduces anti-viral responses of the immune system^13^.

Previous research has demonstrated that co-exposure to oil and hypoxia impacts developing *C. variegatus* as well as *Fundulus grandis*^14–17^ and can reduce egg production and fertilization in adults^18^. Despite the fact that both oil and hypoxia are known to affect developing *C. variegatus*, and that both stressors are known to affect fish immune systems, little is known about how these two stressors interact to impact immune system development. This is an important question as wild fish are rarely only exposed to a single stressor. Understanding the impacts of oil and hypoxia on the developing immune system is, therefore, key to determining the realistic effects of an oil spill such as the *Deepwater Horizon* event. Unfortunately, using traditional assays to assess immune function in larval fish species is difficult, as the small size precludes collection of blood or plasma, and many of the antibodies that would be used to detect proteins display poor cross-reactivity across species, limiting their utility.

The goal of this research was to understand how both stressors (oil and hypoxia) alone and in combination impact the immune system of developing *C. variegatus* by using RNA sequencing (RNAseq) to assess the expression of immune-related genes and pathways in three early life stages. We exposed *C. variegatus* to oil and/or hypoxia for 48 hours during embryonic, post-hatch, or post-larval life stages. We then performed RNAseq and bioinformatic analyses to determine differentially expressed transcripts and pathways between treatment groups and age stages, with a specific focus on immune-related genes and pathways. Previous research in developing fish has found that exposure to oil does significantly impact some immune-related pathways^19–21^. Therefore, we wanted to examine how the effects of these stressors change transcriptional patterns as the developing fish undergo two significant transitions: between the embryonic and post-hatch life stages, and between the post-hatch and post-larval life stages. This study provides novel insight into how immune-related genes and pathways change over the course of development and how exposure to oil and/or hypoxia alters those immune-related genes and pathways. We hypothesized that different life stage transitions would exhibit different transcriptional immune signatures and that the addition of oil and/or hypoxia would modulate these transcriptional immune signatures during development in *C. variegatus*.

## Materials and Methods

### Fish culture and breeding

Sheepshead minnows (*C. variegatus*) were chosen for this research as they are small estuarine fish found throughout the Gulf of Mexico and are both consistently exposed to hypoxic conditions and plausibly threatened by oil released from the *Deepwater Horizon* spill. Adult sheepshead minnows from The University of Southern Mississippi’s Gulf Coast Research Laboratory broodstock (initiated from uncontaminated sites and maintained for greater than 10 generations in clean laboratory conditions) were used to obtain embryos for these experiments. Embryos were collected following standard protocols^15^. All *C. variegatus* individuals were treated humanely in accordance with The University of Southern Mississippi’s (USM) approved IACUC regulations (approved IACUC#: 08110422; approved by USM). All methods were carried out using USM’s approved guidelines and regulations.

### Experimental design

Four treatment groups were used for each life stage (4 replicates per treatment and 20 fish/replicate). Treatments consisted of: 1. Normoxic control, 2. Hypoxic control, 3. Normoxic oil, and 4. Hypoxic oil. Normoxia was defined as dissolved oxygen levels >5.0 mg/L O_2_, and hypoxia as <2.75 mg/L O_2_. Three life stages were tested and were defined by the following start times: embryonic (≤12 hours post-fertilization, hpf), post-hatch (≤12 hours post-hatching, hph), and post-larval (96 hph, after depletion of the yolk-sac and transition to free feeding). Oil treatments were made using a High Energy Water Accommodated Fraction (HEWAF) oil mixture (concentrations ranged from 69.79–168.05 µg/L; exact concentrations for each life stage and treatment may be found in Table S1A). As a reference, sampling operations for depths above 20 m after the *Deepwater Horizon* oil spill observed total polycyclic aromatic hydrocarbon concentrations for 50 PAHs (TPAH50) of up to 240 µg/L^22^. For all life stages (embryonic, post-hatch, and post-larval), experiments were conducted as static, 48-hour exposures at 30 °C and 30 ppt salinity with a 16:8 light:dark cycle. After termination of each experiment, ten fish from each treatment were pooled, in quadruplicate, and placed into Ambion RNAlater solution (ThermoFisher, Waltham, MA, USA) and stored at −80 °C until further analysis.

### HEWAF preparation

HEWAF was prepared using previously established methods^23,24^. Briefly, HEWAF stock was created by using crude, source oil collected from the riser pipe during the *Deepwater Horizon* oil spill at a ratio of 1 g oil per L of artificial seawater (salinity 30 ppt) in a stainless steel blender (Waring, Stamford, CT, USA). The mixture was then blended using the low speed setting for 30 s and the water/oil mixture was transferred to a 2L separatory funnel, covered with foil, and allowed to settle for 1 hr. Next, the dissolved fraction of oil was diluted to the appropriate concentration (12.5%) and used in the oil-exposure treatments.

### Water chemistry

HEWAF subsamples from stock solutions and subsamples from each exposure container were collected and analyzed for PAH composition. For each stock, 250 mL of water was collected, placed in an amber glass bottle, and refrigerated at 4 °C before shipment to The Center for Environmental Sciences and Engineering (Storrs, Connecticut) where they were analyzed via gas chromatography coupled with tandem mass spectrometry (GC/MS/MS). Every sample was analyzed for 29 PAH analytes to quantify total PAHs in solution. Financial costs precluded a full GC/MS/MS analysis of every replicate. To examine within and between treatment variability, subsamples were analyzed via fluorescence using the SpectraMax M2 spectrometer (Molecular Devices, San Jose, CA, USA) using previously published methods^25^. Briefly, test water (3.5 mL) was collected from each replicate at 0 h, 24 h, and 48 h. The water samples were mixed 1:1 with ethanol and stored in 7 mL scintillation vials at 4 °C until measurement. Total PAHs were quantified by measuring fluorescence between 270 and 380 nm^26^. See Table S1A for water chemistry and S1B for a list of PAH analytes measured.

### RNA isolation and sequencing

Total RNA was isolated from 10 fish from each treatment, in triplicate, following the manufacturer’s instructions using an RNeasy kit (Qiagen, Hilden, Germany). RNA quantity and integrity were assessed via a NanoDrop 2000 Spectrophotometer (ThermoFisher, Waltham, MA, USA) and an Agilent 2100 Bioanalyzer (Agilent, Santa Clara, CA, USA). Once samples were determined to be of appropriate quality and quantity (RIN scores of ≥6), total RNA was sent to Purdue University’s Genomics Core Facility (West Layfette, IN, USA) for cDNA library preparation and sequencing. Illumina TruSeq Stranded mRNA Sample Preparation Guide (Illumina, San Diego, USA) was used to construct polyA+ libraries, and an Illumina HiSeq 2500 was used to sequence 2 × 100 bp sequencing reads with a minimum depth of 30 million reads/sample. Table S2 contains additional information about read counts and mapping for each sample.

### Bioinformatics analysis

**Figure 1:**
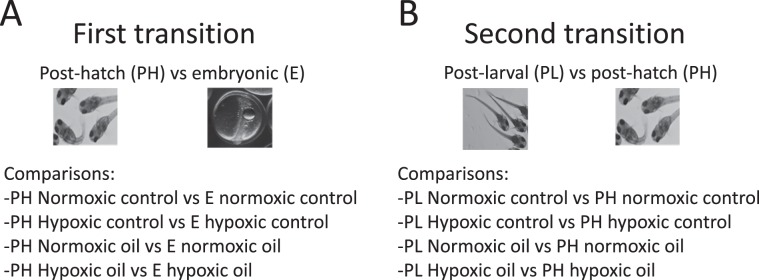
Representation of comparisons examined in the first life stage transition (**A**) and second life stage transition (**B**). Photographs taken by Danielle Simning.

CLC Genomics Workbench (Qiagen, Hilden, Germany) was used for adaptor trimming and to remove failed reads and reads with low quality scores and obtain a mean Phred score of 30. Paired end reads were then merged and mapped to the *C. variegatus* reference genome (NCBI accession number: GCF_000732505.1) using CLC. Transcripts per million (TPM) were calculated in CLC and differential gene expression values were calculated. A total of 8 analyses were run to examine changes in the expression of immune related genes with age and how oil and/or hypoxia altered this expression. The 8 analyses were: 1. post-hatch normoxic control vs. embryonic normoxic control, 2. post-hatch hypoxic control vs. embryonic hypoxic control, 3. post-hatch normoxic oil vs. embryonic normoxic oil, 4. post hatch hypoxic oil vs. embryonic hypoxic oil, 5. post-larval normoxic control vs. post hatch normoxic control, 6. post-larval hypoxic control vs. post-hatch hypoxic control, 7. post-larval normoxic oil vs. post-hatch normoxic oil, and 8. post-larval hypoxic oil vs. post-hatch hypoxic oil (Fig. 1). Effects from treatments within each life stage are outside of the scope of this manuscript and will be discussed in future work. As we were primarily interested in how oil and hypoxia modulated the normal expression on immune genes as the fish developed from one life stage to the next, the post-hatch vs. embryo comparison is not included.

Differentially expressed genes (DEGs) were sorted by FDR p-value, and genes from each comparison were converted into their human orthologs and analyzed using Ingenuity Pathway Analysis (IPA) (Qiagen, Hilden, Germany). Pathway analyses were based on expression fold change and the FDR p-value cutoff was set at 0.05. In order to focus on immune related-pathways, four immune-related categories were selected for inclusion: Cellular Immune Response, Cytokine Signaling, Humoral Immune Response, and Pathogen-Influenced Signaling, comprising 115 possible immune-related pathways. In addition, we limited our pathway analysis to only include pathways that had a predicted directionality associated with them. With our 8 analyses, we found a total of 48 significant, unique immune pathways represented. All genes involved in all the aforementioned immune-related pathways (a total of 615 genes from the 48 significant pathways) were used to examine the effects of age stage progression, oil, and hypoxia exposure on transcriptional regulation of immune pathways in developing *C. variegatus*.

**Figure 2:**
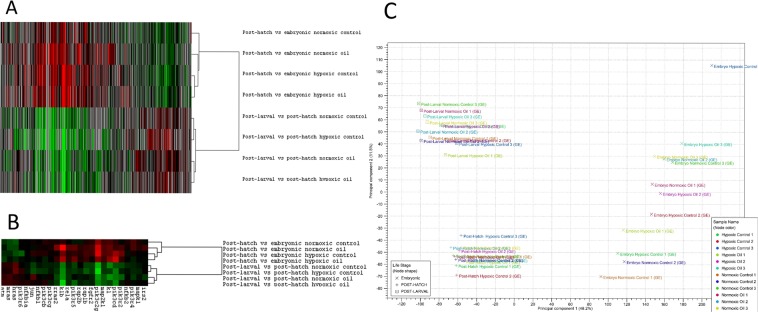
Hierarchical clustering for all genes represented in any immune-related pathway (**A**), and for the top 25 most common immune-related genes from all pathways (**B**) generated in Java TreeView software version 1.1.6r4 (http://jtreeview.sourceforge.net/). Principal component analysis for all samples (**C**) generated in CLC Genomics Workbench 11 (https://www.qiagenbioinformatics.com/products/clc-genomics-workbench/).

**Figure 3:**
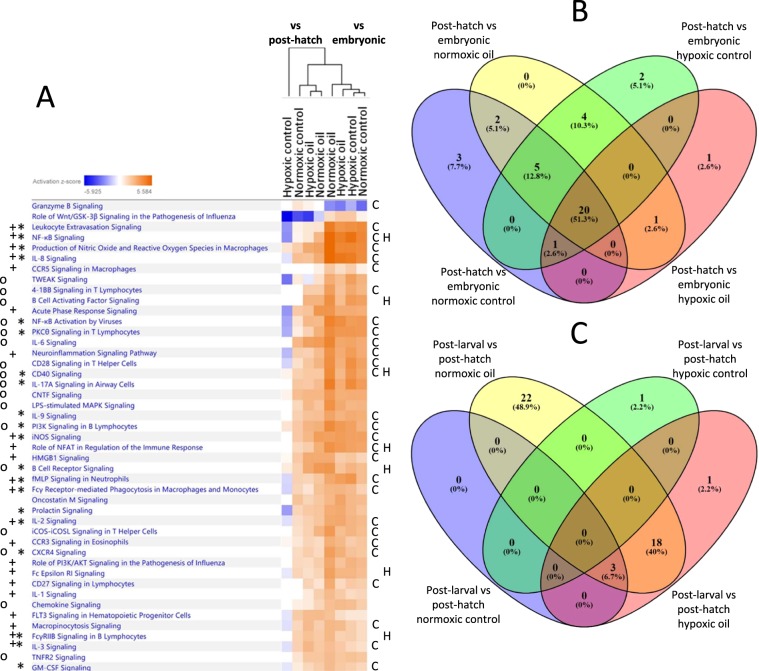
Hierarchical clustering of immune-related canonical pathways of all treatment groups in the first and second life stage transitions,* indicates a pathway shared between all treatment groups in the first life stage transition, +indicates a pathway unique to the normoxic oil group in the second life stage transition, O indicates a pathway shared between normoxic oil and hypoxic oil groups in the second life stage transition, C indicates a cellular immune response pathway, and H indicates a humoral immune response pathway (**A**). (**A**) was generated in IPA (no version number) (https://www.qiagenbioinformatics.com/products/ingenuity-pathway-analysis/). Comparison of number of significantly regulated immune-related pathways in the first (**B**) and second (**C**) life stage transitions for each treatment. (**B**,**C**) were generated in Venny 2.1.0 (https://bioinfogp.cnb.csic.es/tools/venny/).

To identify the impacts of the different treatments, we analyzed the data in two ways. First, we examined the transcriptional patterns of the genes in the selected pathways to identify patterns of clustering that were informative of treatment effects. Second, we identified the canonical pathways that were affected in terms of which pathways were most statistically significantly different, and in terms of which pathways were most activated or suppressed in different life stage transitions under exposure to oil, hypoxia, or both. To perform hierarchical clustering (Fig. 2), Gene Cluster 3.0 software (Stanford University, Palo Alto, CA, USA) and Java TreeView software version 1.1.6r4 (http://jtreeview.sourceforge.net/) were used. In Gene Cluster 3.0, gene and pathway data (based on –log p-values) were centered around the mean, clustered based on correlation (uncentered) and linked via average linkage. Data were then entered into Java TreeView visualization, and this software was used to generate Fig. 2A,B. CLC was used to construct the principal component analysis in Fig. 2C. To construct Venn diagrams in Fig. 3B,C, Venny 2.1.0 was used^27^. All significant immune-related pathways from all treatment groups were used for construction of two Venn diagrams, one for each life stage transition comparison. Table S3 contains the full list of significant pathways identified from each treatment. The heat map presented in Fig. 3 was generated by IPA using activation z-scores, which are weighted using underlying findings, relationship bias, and dataset bias. Figure S1 was generated using IPA. All immune pathways were also used for comparison analyses: pathways were sorted by total absolute –log p-value (adding the treatments together for each pathway).

## Results

HEWAF PAH concentrations for stock and experimental solutions are found in Table S1A, and a list of the 29 compounds measured can be found in Table S1B. Mean ± SD of dissolved oxygen was 6.39 ± 0.04 mg/L, 5.33 ± 0.09 mg/L, and 5.12 ± 0.05 mg/L for embryonic, post-hatch, and post-larval normoxic groups, respectively. For the hypoxic groups, dissolved oxygen was 2.45 ± 0.05 mg/L, 2.52 ± 0.05 mg/L, and 2.64 ± 0.07 mg/L for embryonic, post-hatch, and post-larval groups, respectively. All temperatures ranged from 28.6 to 29.3 °C with salinity ranging from 29.9 to 32.4 ppt. Only fish that survived the exposure were used for RNA sequencing. Embryos displayed a mortality of ~15–25%, post-hatch fish ~5–15%, and post-larval fish ~50–80%: for more information on mortality and environmental parameters, see Simning *et al*. 2019 (ref. ^15^).

Hierarchical clustering of total immune gene expression data (Fig. 2A) and gene expression data from the top 25 most commonly occurring immune-related genes from our data (Fig. 2B) showed that life-stage transition clearly clustered differential gene expression into two groups: the first life stage transition (from embryo to post-hatch) and the second life stage transition (from post-hatch to post-larval). Within the first life stage transition, both hypoxic groups clustered together. In the second life stage transition, there was an apparent effect of oil exposure on gene expression clustering, as both normoxic and hypoxic oil treatments were separated from the normoxic and hypoxic control treatments. This pattern was found both in the all genes dataset (Fig. 2A), and in the top 25 genes dataset (Fig. 2B), indicating that the transcriptional response was not solely driven by a handful of highly affected genes, but was a consistent pattern across the immune transcriptome. A principal component analysis (PCA) revealed that individual samples clustered within their respective life stages, supporting the hierarchical clustering finding of differentially expressed genes clustering by age (Fig. 2C).

We next classified the genes into their respective pathways to identify patterns in altered immune responses as a function of age stage transition and treatment. We examined the relationships between pathway activation status (measured by z-score) and treatment using hierarchical clustering (Fig. 3A). Here, as in the gene expression response data, we found that the two transitions had consistently divergent responses, and that treatment effects were nested within transition effects. In the first transition, the two control treatments (normoxic and hypoxic) were most similar to each other, with hypoxic and normoxic oil clustering next, indicating that these treatments produced responses that were different from control, and each other. Within the second (post-hatch to post-larval) transition, there was some evidence of an oil-driven structuring to the response pattern, as both hypoxic and normoxic oil produced similar responses, with normoxic controls clustering separately; a pattern also observed in the hierarchical clustering of immune genes. Interestingly, the hypoxic control from this transition clustered away from all other treatments, indicating that it produced a response in immune pathway activation status that was highly divergent from the others, a response not observed in the gene expression data. Overall, there was a general pattern of pathway activation in all treatment conditions except for the hypoxic control treatment in the second transition, which showed a general pattern of pathway suppression. Even though most pathways were activated in most treatments, “Granzyme B Signaling” in the first age transition, and “Role of Wnt/GSK-3β Signaling in the Pathogenesis of Influenza” in the second transition, were both suppressed under all exposure scenarios.

In examining the pathways that were affected during each of the two transitions, interesting patterns emerge. During the first life stage transition, the transcription of 31 immune-related pathways were activated under control conditions as the fish moved from embryos to post-hatch larvae (Fig. 3B, sum of all cells contained under “Post-hatch vs embryonic normoxic control”). This may be considered as the normal development of immune pathways in developing *C. variegatus* during this transition. The addition of oil and hypoxia as stressors at this life stage transition had a minimal effect on the number of immune pathways that displayed altered activation status. A total of 35 immune-related pathways were identified as significantly affected between embryos and yolk-sac larvae in at least one stressor treatment (Fig. 3B), but of these, 27 pathways (77%) were also affected in the control transition, and 20 were activated under all exposure conditions (Fig. 3A, indicated by *). This indicates both that this is a time during which the activity of immune pathway transcription is tightly constrained, and that these pathways were relatively resistant to oil and/or hypoxia-induced dysfunction. The majority of the shared pathways (16/20, 80%) that were activated under all exposure scenarios are related to cellular immune responses. In addition, half of these pathways are related to cytokine signaling (note that there is overlap in pathway membership). As cellular immune responses are critically important for fishes, and considering the wide variety of function performed by cytokines, it is not surprising that these types of pathways are actively regulated at this early life transition.

During the second life stage transition from post-hatch larvae to post-larval fish, only 3 immune related pathways were altered under normoxic control conditions, indicating that under normal environmental circumstances there is relatively little alteration of immune pathways (Fig. 3C). In contrast to the first transition, there was evidence of treatment-related effects on the activation status of immune pathways in developing fish larvae. A total of 42 immune-related pathways were significantly affected by at least one non-control normoxia exposure scenario, almost entirely as a result of oil exposure. Under normoxic oil exposure, 43 pathways were significantly affected in this life stage transition, including 3 that were altered under control conditions, 22 that were unique to that exposure scenario (Fig. 3A, indicated by +), and 18 that were also affected under hypoxic oil exposure (Fig. 3A, indicated by O). Neither hypoxia alone nor hypoxia+oil had a distinct effect on significant pathways, indicating that oil exposure was the primary factor driving effects on immune pathways in the second developmental transition.

The set of 22 pathways that were only affected under normoxic oil exposure in the second transition (Fig. 3A, +) were similar to the set of 20 pathways universally activated in the first life stage transition (Fig. 3A, *). Ten pathways were activated under both conditions, potentially indicating that oil exposure during the second transition affects many of the same immune processes that are normally developing during the first transition. These 10 pathways were mostly related to cellular immune responses (9 of 10) and cytokine signaling (5 of 10, all of which overlapped with cellular immune response-related pathways), though two pathways were also associated with humoral immune responses. One of the significantly impacted pathways, “B Cell Receptor Signaling”, is highlighted in Fig. S1. Of the 18 pathways that were activated in the second age transition under both normoxic and hypoxic oil exposure scenarios (Fig. 3A, O), 8 were related to cellular immune responses and 8 were related to cytokine signaling, indicating that these pathways may represent a unique oil-driven immune response, regardless of oxic regime, in this transition.

To more fully understand the potential impacts of the tested stressors, we next classified the immune pathways into the general categories of cellular and humoral immune responses. We did not specifically target innate and adaptive immune responses because fish immunity is largely innate in nature, and adaptive immune responses are likely not fully developed in these early life stages. All immune pathways impacted in any treatment or life stage were binned into the broader categories based on their functionalities (e.g. “B Cell Receptor Signaling” was categorized as a humoral immune response pathway); some pathways (e.g. “Prolactin Signaling”) were excluded for being neither truly cellular or humoral in nature, while other pathways (e.g. “NF-κB Signaling”) have such broad functionalities that they were placed in both cellular and humoral response categories (Fig. 3A, C for cellular immune response pathway, H for humoral immune response pathway).

**Figure 4:**
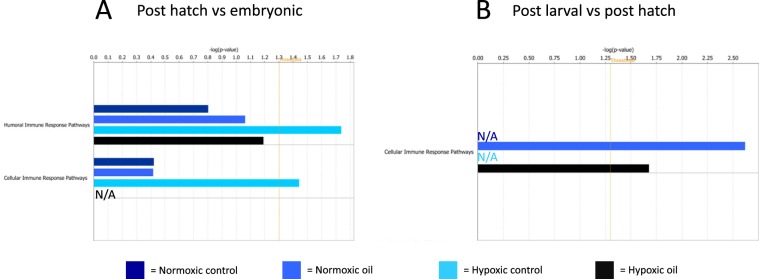
Cellular and humoral immune responses in the first (**A**) and second (**B**) life stage transitions. Note that humoral immune response values are not shown in part B as there were no significant data. Both figures were generated in IPA (no version number) (https://www.qiagenbioinformatics.com/products/ingenuity-pathway-analysis/).

Overall patterns of cellular and humoral immune response for each treatment in both life stages are visualized in Fig. 4. For the first life stage transition, humoral immune response were elevated in the hypoxic control group only (Fig. 4A). In addition, cellular immune responses were only significantly impacted in the hypoxic control treatment in this transition; impacts that are non-existent when oil is present in conjunction with hypoxia—indicating a possible effect of hypoxia that is abolished by the addition of oil. In the second life stage transition, humoral immune responses were not significantly impacted in any treatment (data not shown), while cellular immune responses were significantly impacted in the oil treatments (normoxic and hypoxic) and not in the non-oil treatments (Fig. 4B). The lack of significance observed in the non-oil treatments for both cellular and humoral immune responses in this second life stage transition is possibly due to the low number of significantly impacted immune-related pathways for these treatments at this life stage transition.

## Discussion

Our data suggest that in the first life stage transition, exposure to oil, hypoxia, or both has only a marginal effect on the regulation of immune pathways in sheepshead minnows. This may be partially due to the fact that the immune system is still undergoing development and during this transition fish are being fully exposed to their environment for the first time, which is likely to contain bacteria, viruses, parasites, and other foreign antigens that induce immune responses and swamp out any potential stressor-induced effects. Conversely, in the second transition, exposure to oil under both normoxic and hypoxic conditions caused significant alteration of many immune pathways (40 in normoxic oil, 22 in hypoxic oil, 18 present in both). These data indicate that early life stage fish display differential susceptibility to contaminant-induced effects on immune pathways; that small age differences can have dramatic effects on immune-related transcriptomic responses to stressors; and that larval fish in the transition from post-hatch (yolk-sac feeding) to post-larval (free feeding) may have increased susceptibility to contaminant-induced immune effects.

Interestingly, the direction of pathway effects observed were almost always activation, except in the hypoxia only treatment in the second life stage transition (Fig. 3A). In the normoxic control exposure scenarios, this may indicate that immune-related pathways are developing and being activated, particularly as the fish transition from embryo to yolk-sac larvae, and then from yolk-sac larvae to free feeding larvae. Research examining mahi mahi (*Coryphaena hippurus*) transitioning from embryos to yolk-sac larvae and from yolk-sac larvae to free-feeding larvae found three immune-related pathways that were activated in both of these life stage transitions: “Role of NFAT in Regulation of the Immune Response” (also activated in our normoxic oil treatment in the second life stage transition), “IL-6 Signaling,” and “fMLP signaling in Neutrophils”^19^, indicating that activation of immune pathways in early life stage fishes is likely part of normal development. One possible explanation for these results is that cortisol levels, known to modulate immune parameters in fishes^28^, tend to increase post-hatching as a routine part of development^29^, which may contribute to the immunomodulation that we observe in the control fish.

One common response present among the altered immune pathways in both life stage transitions was the role of macrophages and phagocytosis (in pathways such as “Production of Nitric Oxide and Reactive Oxygen Species in Macrophages,” “Fcγ Receptor-mediated Phagocytosis in Macrophages and Monocytes,” and “CCR5 Signaling in Macrohpages”; pathways found in every treatment in the first life stage transition and in the normoxic oil treatment only in the second life stage transition). Macrophages and monocytes are important immune accessory cells in fishes that are involved in cellular immune response pathways and play a critical role in responses to T cell stimuli^30^. Previous research in adult sheepshead minnows exposed to oil observed that immune pathways and processes were impacted, with impacts to macrophages (infiltration, apoptosis, polarity, and activation) occurring after seven days of exposure^31^. Other research suggests that phagocytosis may be impacted by exposure to some PAHs in some fish species: for example, BaP and benzo[a]anthracene (BaA) decreased phagocytosis in rainbow trout (*Oncorhynchus mykiss*)^32^, but BaP did not decrease phagocytic activity in sea bass (*Dicentrarchus labrax*)^33^ or tilapia (except at a dose high enough to reduce feeding and increase mortality)^34,35^. Another prevalent pathway tied to macrophages is that surrounding respiratory burst and the production of reactive oxygen species. Respiratory burst in fish phagocytes after exposure to PAHs can be enhanced^33,36,37^ or suppressed^34,35,38–40^, depending on fish species, route of exposure, dosage, and specific type of phagocyte^41^. These studies highlight that cellular immune response pathways in fishes are significantly impacted by PAH exposure: a finding that was also present in our oil-exposed treatments during the second life stage transition. However, the effects on cellular immune response pathways are complex and are linked to many factors surrounding the exposure itself.

Another common response in the altered immune pathways in both life stage transitions was related to cytokine signaling, which is likely due to the prevalent and varied nature of cytokines and their functions. Previous research in juvenile red snapper (*Lutjanus campechanus*) found that several cytokines were upregulated after eight days of oil exposure: interleukin 10 (*il10*), *il8*, and *il1β*^42^. Research in juvenile Japanese flounder (*Paralichthys olivaceous*) showed an upregulation of *il8* after two days of heavy oil exposure, even if fish were also allowed to depurate for five days after exposure^43^, signaling that PAHs do impact cytokine production and expression, even in juvenile fishes.

Several of our findings correspond with previous research in mahi mahi (*C. hippurus*) at two life stage transitions (the first from 24 to 48 hours post-fertilization, hpf, and the second from 48 to 96 hpf)^19^. Our study found significant activation of “Role of NFAT in Regulation of the Immune Response” in three of four first life stage transition treatments and the normoxic oil second life stage transition treatment, significant regulation of “IL-6 Signaling” in two first life stage and two second life stage transition treatments, and significant regulation of “fMLP Signaling in Neutrophils” in all treatments in the first life stage transition and one treatment in the second life stage transition. The mahi mahi study noted activation of these same three pathways in both life stage transitions^19^. Interestingly, magnitude and directionality of immune-related transcriptional responses appear to depend on length of oil exposure and type of oil: most immune-related pathways were activated at 96 hours of exposure to slick oil in mahi mahi, but were mostly unaffected at 96 hours of exposure to source oil^20^. At 48 hours of exposure to source of slick oil (the same amount of time as our exposure duration), most immune pathways were suppressed or unchanged in mahi mahi, and at 24 hours of exposure to source or slick oil, no immune pathways were altered^20^.

Hypoxia is also known to affect the immune system of fish, and we found that hypoxia alone significantly alters cellular and humoral immune response pathways in the first life stage transition, but not the second life stage transition. Previous research highlights that fish experiencing hypoxic conditions can have decreased complement activity^9^, reduced production of reactive oxygen species^10^, depressed respiratory burst activity in head kidney leukocytes^11^, and lower antibody responses^12^. Though hypoxia can clearly impact the fish immune system, our results suggest that timing of hypoxia exposure matters: experiencing hypoxia during the first life stage transition (embryo to post-hatch) significantly impacts a greater number of immune-related pathways (32) as well as overall cellular and humoral immune responses. Though this pattern is not observed in the second life stage transition, hypoxia has a unique signature of generally deactivating immune pathways, which is a pattern that is not observed in the first life stage transition.

Overall, our data suggest that life stage plays a major role in regulation of immune genes and pathways and that life stage influences the response to hypoxia and/or oil exposure. These data suggest that even small differences in age profoundly impact the immune system in early life stage fishes, and that future work should strongly consider which life stage(s) are the most relevant for the question(s) being asked, as each life stage has unique responses. While our data are based solely on bioinformatic analyses of gene expression, not traditional immune assays, these data demonstrate that immune genes are transcriptionally active in early life stage fish, that the expression patterns differ between developmental stages that are very close in age, that exposure to exogenous stressors can have a definable impact on the transcription of genes involved in immune pathways, and that modern sequencing and bioinformatic approaches can be useful to assess the effects of stressors on the immune system of developing fish, when the traditional immune assays are not possible.

An important area for future studies includes investigating the confounding effect(s) that other environmental factors (such as temperature) may have on immune system development, especially in conjunction with other stressors, such as a bacterial or viral exposure. The immune system it not only crucial to organisms for its ability to fight off foreign invaders, but the immune system and its responses also have overlap and downstream consequences for other bodily responses. Approaches examining immune-related responses under real-world multistressor scenarios will aid in fully understanding the impacts of a variety of toxicological stressors on overall organismal health and survival, and will also allow for a deeper understanding of the effects of environmental disasters, such as the *Deepwater Horizon* oil spill.

## Supplementary information

Supplementary information.

Supplementary information 2.

Supplementary information 3.
