# Publisher Correction: Exposure to Oil and Hypoxia Results in Alterations of Immune Transcriptional Patterns in Developing Sheepshead Minnows (*Cyprinodon variegatus*)

**DOI:** 10.1038/s41598-020-61186-w

**Published:** 2020-03-11

**Authors:** Maria L. Rodgers, Danielle Simning, Maria S. Sepúlveda, Sylvain De Guise, Thijs Bosker, Robert J. Griffitt

**Affiliations:** 1 Division of Coastal Sciences, School of Ocean Science and Engineering, University of Southern Mississippi, Ocean Springs, Mississippi 39564 USA; 2 Purdue University, Department of Forestry and Natural Resources, West Lafayette, Indiana, 47907 USA; 3 Department of Pathobiology and Veterinary Science, University of Connecticut, Storrs, Connecticut 06269 USA; 4 Leiden University College/Institute of Environmental Sciences (CML), Leiden University, Leiden, 2595DG The Netherlands

Correction to: *Scientific Reports* 10.1038/s41598-020-58171-8, published online 03 February 2020

The original version of this Article contained an error in the email address given for the author Robert J. Griffitt. The correct email address is given below:

joe.griffitt@usm.edu

Additionally, this Article contained a truncated version of Figure 3.

These errors have been fixed in the HTML and PDF of the original Article.

