# Supplementary material for: Exposure to Oil and Hypoxia Results in Alterations of Immune Transcriptional Patterns in Developing Sheepshead Minnows (Cyprinodon variegatus)
Source: Sci Rep. 2020 Feb 3;10:1684. doi: 10.1038/s41598-020-58171-8 (PMC6997411; doi:10.1038/s41598-020-58171-8)
Supplement: Supplementary file 1 — Supplementary information. [file 41598_2020_58171_MOESM1_ESM.pptx]

## Slide 1
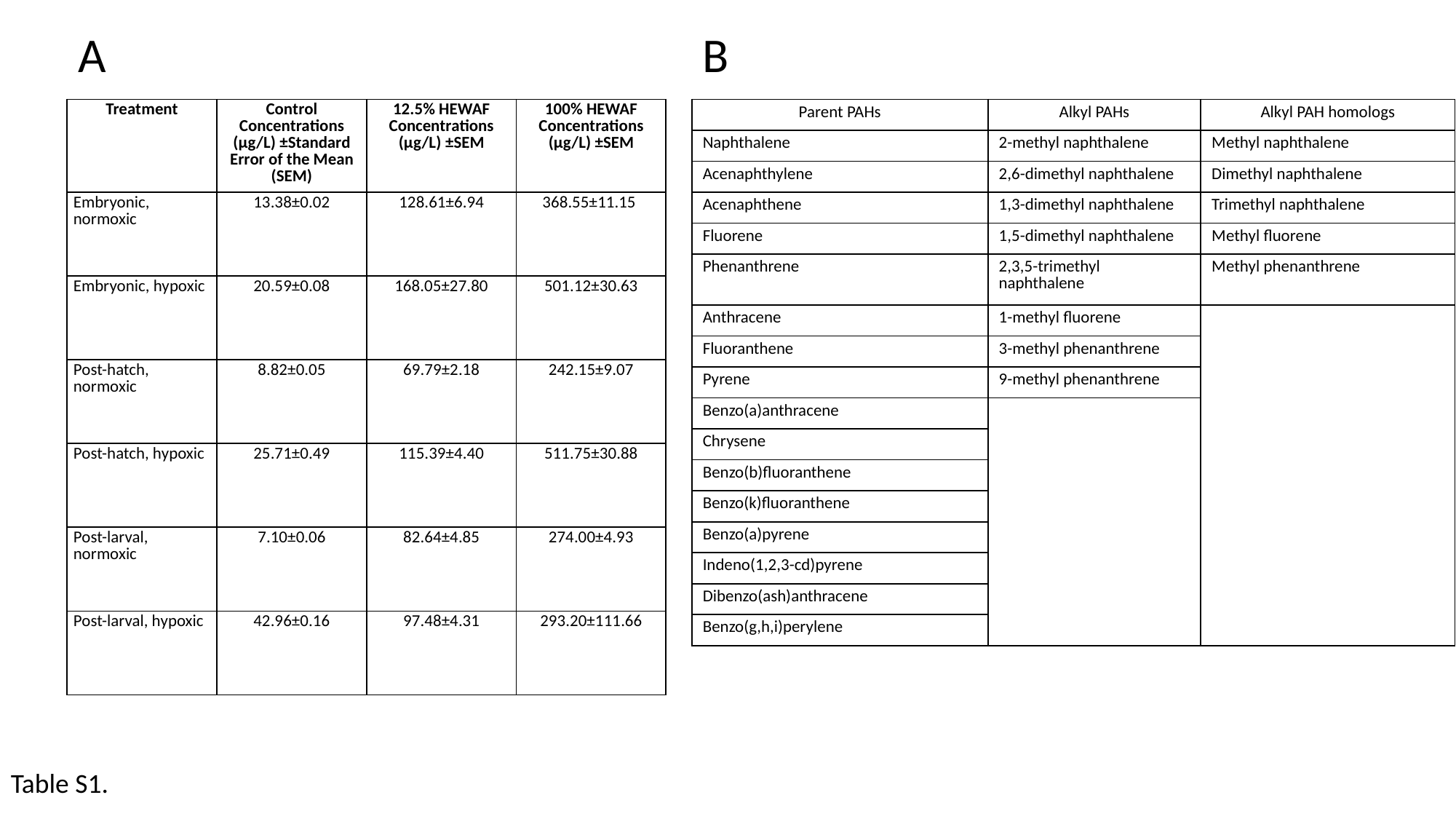

A
B
| Treatment | Control Concentrations (µg/L) ±Standard Error of the Mean (SEM) | 12.5% HEWAF Concentrations (µg/L) ±SEM | 100% HEWAF Concentrations (µg/L) ±SEM |
| --- | --- | --- | --- |
| Embryonic, normoxic | 13.38±0.02 | 128.61±6.94 | 368.55±11.15 |
| Embryonic, hypoxic | 20.59±0.08 | 168.05±27.80 | 501.12±30.63 |
| Post-hatch, normoxic | 8.82±0.05 | 69.79±2.18 | 242.15±9.07 |
| Post-hatch, hypoxic | 25.71±0.49 | 115.39±4.40 | 511.75±30.88 |
| Post-larval, normoxic | 7.10±0.06 | 82.64±4.85 | 274.00±4.93 |
| Post-larval, hypoxic | 42.96±0.16 | 97.48±4.31 | 293.20±111.66 |
| Parent PAHs | Alkyl PAHs | Alkyl PAH homologs |
| --- | --- | --- |
| Naphthalene | 2-methyl naphthalene | Methyl naphthalene |
| Acenaphthylene | 2,6-dimethyl naphthalene | Dimethyl naphthalene |
| Acenaphthene | 1,3-dimethyl naphthalene | Trimethyl naphthalene |
| Fluorene | 1,5-dimethyl naphthalene | Methyl fluorene |
| Phenanthrene | 2,3,5-trimethyl naphthalene | Methyl phenanthrene |
| Anthracene | 1-methyl fluorene | |
| Fluoranthene | 3-methyl phenanthrene | |
| Pyrene | 9-methyl phenanthrene | |
| Benzo(a)anthracene | | |
| Chrysene | | |
| Benzo(b)fluoranthene | | |
| Benzo(k)fluoranthene | | |
| Benzo(a)pyrene | | |
| Indeno(1,2,3-cd)pyrene | | |
| Dibenzo(ash)anthracene | | |
| Benzo(g,h,i)perylene | | |
Table S1.

## Slide 2
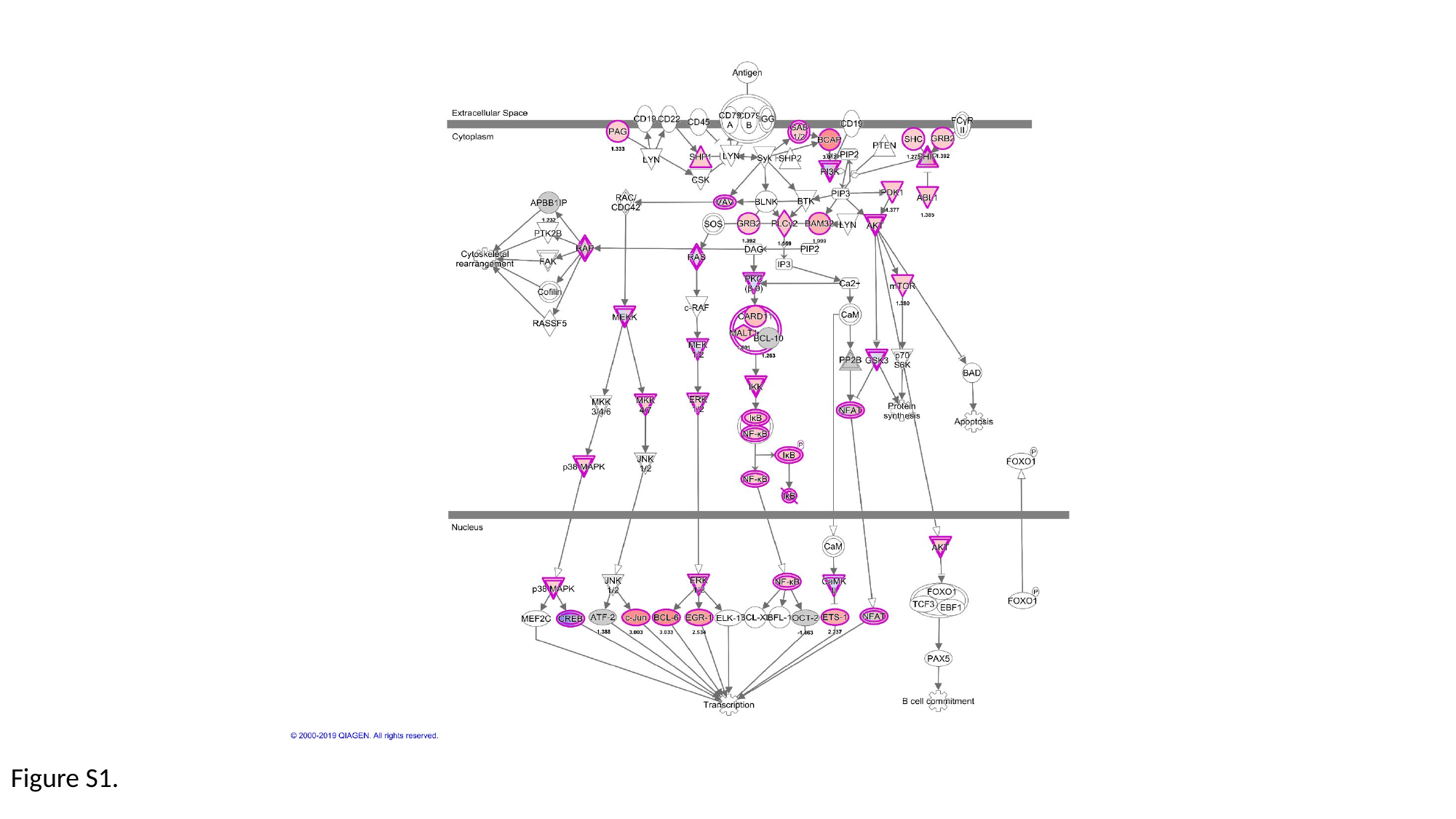

Figure S1.
